# The bidirectional effects and mechanisms of the oral and gut microbiomes: a narrative review

**DOI:** 10.3389/fimmu.2026.1697413

**Published:** 2026-02-23

**Authors:** Tiantian Huo, Xiaofei Huang, Jiayi Liao, Huo Zhang, Li Hu, Mengru Xie

**Affiliations:** 1 Department of Stomatology, Union Hospital, Tongji Medical College, Huazhong University of Science and Technology, Wuhan, China; 2 School of Stomatology, Tongji Medical College, Huazhong University of Science and Technology, Wuhan, China; 3 Hubei Province Key Laboratory of Oral and Maxillofacial Development and Regeneration, Wuhan, China; 4 Key Laboratory of Molecular Biophysics of Ministry of Education, College of Life Science and Technology, Huazhong University of Science and Technology, Wuhan, China; 5 Department of Orthodontics, Affiliated Stomatology Hospital of Guangzhou Medical University, Guangzhou, Guangdong, China

**Keywords:** gut microbiome, oral microbiome, periodontal disease/periodontitis, Porphyromonas gingivalis, systemic health/disease

## Abstract

Among the microbial ecosystems of the human body, the gut and oral microbiota constitute the two largest communities, collectively harboring thousands of bacteria, fungi, and viruses. Under physiological conditions, these microbiotas maintain internal homeostasis and stability, thereby protecting the host against pathogenic colonization. However, when pathogens such as *Porphyromonas gingivalis* translocate from the oral cavity to the gut, disruption of gut microbial homeostasis may occur, increasing the risk of disease development. Potential mechanisms underlying this association include the establishment of new symbiotic relationships, the disruption of the intestinal barrier, the activation or suppression of inflammatory cells—particularly the balance between T helper 17 (Th17) cells and regulatory T cells (Tregs)—and the induction of systemic inflammation. Conversely, gut microbiota dysbiosis, as observed in patients with inflammatory bowel disease, irritable bowel syndrome (IBS), or colorectal cancer, is also associated with alterations in the composition and diversity of the oral microbiota. Factors such as immune cell migration, malnutrition, and taste disturbances may contribute to oral microbial imbalance. In this review, we summarize the bidirectional influences on the composition and diversity of the oral and gut microbiomes and propose potential mechanisms underlying their interactions. A deeper understanding of these processes will enhance our knowledge of microbiota–host interactions and systemic health, and may shed light on the prevention and treatment of systemic diseases related to oral and gut microbiota dysbiosis.

## 1 Introduction

The number of microorganisms colonizing the external and internal environments of the human body is estimated to be at least ten times greater than the number of human cells (1). These microorganisms are essential for normal physiological function. Among them, the gut and oral microbiomes represent the two largest microbial ecosystems in the human body (1). According to the Human Microbiome Project, more than half of the body’s bacteria are located in the oral cavity (26%) and the gastrointestinal tract (29%) (1). In recent years, increasing attention has been given to the relationship between the oral and gut microbiota. Under both healthy and pathological conditions, these two microbial communities interact and exert reciprocal influences. The oral cavity is a vital element of the digestive system, and the phenomenon of oral microbiome dissemination and colonization in the gut is common (2). Consistent with this observation, the Human Microbiome Project reported that in more than 200 healthy adults, approximately 45% of bacterial taxa were shared between the oral cavity and fecal samples (1).

Alterations in the oral microbiota, such as those observed in patients with periodontitis or dental caries, are associated with concomitant changes in the composition of the gut microbiota. Epidemiological studies have consistently shown that individuals with oral diseases are more susceptible to intestinal disorders. In particular, tooth loss and periodontitis are strongly associated with inflammatory bowel disease (IBD)–related morbidity and functional impairment (3). Similarly, compared with individuals with a healthy oral cavity, patients with periodontal disease exhibit a 21% increased risk of developing colorectal cancer (CRC) (4). Oral lesions may act as initiating factors in intestinal microbial dysbiosis and may also serve as clinical manifestations of underlying intestinal disease. Periodontal disease is significantly more prevalent in IBD patients, with severe forms occurring more frequently (5). Consistent with these clinical observations, animal studies have shown that mice with Dextran Sulfate Sodium (DSS)-induced colitis exhibit aggravated alveolar bone resorption, indicating that intestinal inflammation can adversely affect periodontal tissues (6). Collectively, these findings suggest bidirectional interactions between the oral and gut microbiota. However, current research primarily focuses on disease outcomes driven by gut microbiota alterations following oral microbial translocation, while the mechanisms by which oral microbiota contribute to gut dysbiosis remain unclear. Based on an integrated overview of the composition and function of the oral and gut microbiota under healthy and diseased conditions, this review examines how perturbations in one microbial community influence the other and explores potential mechanisms underlying their interaction. Ultimately, we aim to identify upstream strategies for restoring gut microbial homeostasis, provide directions for further research on oral–gut microbial interactions, and establish a theoretical basis for managing intestinal and other systemic diseases through targeted treatment of oral disorders.

## 2 Methods

In this review, we aimed to explore the interactions between the gut and oral microbiomes, particularly in the state of oral diseases such as periodontitis and intestinal diseases such as IBD. To ensure a comprehensive and unbiased approach, we conducted a systematic literature search using two major databases: PubMed/NCBI (National Center for Biotechnology Information, US National Library of Medicine) and Web of Science (Clarivate™). We limited our search to original studies published between 2015 and 2026, excluding commentaries and conference abstracts. In PubMed, the following search strategy was applied: (“Periodontitis”[Mesh]. OR “Mouth”[Mesh]) AND (“Microbiota”[Mesh] OR “Bacteria”[Mesh] OR “Fungi”[Mesh] OR “Viruses”[Mesh] OR “Dysbiosis”[Mesh]) AND (“Inflammatory Bowel Diseases”[Mesh] OR “Colorectal Neoplasms”[Mesh] OR “Irritable Bowel Syndrome”[Mesh] OR “Crohn Disease”[Mesh] OR “Colitis, Ulcerative”[Mesh] OR “Gastrointestinal Microbiome”[Mesh]). This search yielded 577 articles. In Web of Science, the search strategy was: “oral cavity” OR periodontitis OR mouth (Topic) and dysbiosis OR microbiome OR fungi OR virus OR bacteria (Topic) and “Inflammatory Bowel Diseases” OR “Colorectal cancer” OR “Irritable Bowel Syndrome” OR “Crohn Disease” OR “Colitis, Ulcerative” OR “Gastrointestinal Microbiome” (Topic) and 2015-2026 (Year Published). This search returned 263 articles. In addition to the database searches, we performed manual screening of references and citation tracking to supplement studies related to therapeutic approaches and clinical applications. The included studies were analyzed qualitatively to provide a comprehensive overview of the pathways and mechanisms linking the oral and gut microbiomes.

## 3 The bidirectional influence between the oral and gut microbiomes

### 3.1 The oral microbiome: functions, dysbiosis, and its impact on gut microbiome

The oral cavity serves as the initial segment of the alimentary canal and hosts the second most abundant microbiota, comprising over 700 bacterial species (7). This community includes major phyla such as *Firmicutes*, *Bacteroidetes*, *Proteobacteria*, *Actinobacteria*, *Fusobacteria*, and *Neisseria (*8), and genera such as *Streptococcus*, *Gemella*, *Veillonella*, *Haemophilus*, *Porphyromonas*, *Fusobacterium*, *Actinomyces*, and *Prevotella (*7). Under physiological conditions, oral microorganisms form a stable symbiotic relationship with the host and perform four key functions: 1) Metabolism. *Fusobacterium* and *Peptostreptococcus* exhibit aminopeptidase activity, converting amino acids into short-chain fatty acids (SCFAs) and other small molecules essential for microbial growth (9). 2) Defense. *Streptococcus dentisani* can inhibit the growth of *S. mutans* and reduce the risk of dental caries by neutralizing plaque acidity and producing hydrogen peroxide (H_2_O_2_), thereby protecting both the biofilm community and the host from infection (10). 3) Influence on host dietary behavior. Certain species of *Clostridia* and *Prevotella* are linked to taste thresholds, and may modulate dietary preferences in ways that favor microbial persistence within the oral cavity (11). 4) Systemic health. The oral microbiome can convert nitrate to nitrite, which is subsequently absorbed into the circulation and converted to nitric oxide (NO), benefit to maintain vascular elasticity and prevent hypertensive effects (12).

The stability and balance of the oral flora are vital to oral health. Disruptions in its composition and distribution can lead to the development of oral diseases. In dental caries, cariogenic bacteria like *Streptococcus*, *Lactobacillus*, and *Bifidobacterium* increase. These organisms metabolize dietary sugars to produce acids that demineralize tooth structure (13, 14). Periodontitis is characterized by chronic inflammation and progressive destruction of tooth-supporting tissues. This condition is primarily associated with an increased abundance of red complex bacteria, including *Porphyromonas gingivalis* (*P. gingivalis*), *Tannerella forsythia*, and *Treponema denticola*. In contrast, beneficial taxa such as *Streptococcus salivarius* are reduced during disease progression (15, 16).

Oral dysbiosis also affects gut microbial balance. Periodontitis patients exhibit reduced alpha diversity of the gut microbiota, accompanied by enrichment of *Porphyromonadaceae*, *Tannerella*, and *Treponema*, and a decrease in *Streptococcaceae*, *Pasteurellaceae*, and *Veillonellaceae* (17, 18). Similar changes have been observed in animal studies. In mice, ligature-induced periodontitis leads to decreased gut microbial alpha diversity and increased abundance of the phylum *Firmicutes* and the genus *Barnesiella* and *Akkermansia* (19). These alterations in the gut microbiota are likely driven by the translocation of oral pathogens under disease conditions. In experimental models, mice gavaged with saliva from periodontitis patients develop disrupted cecal microbiota and low-grade inflammation, marked by increased *Porphyromonadaceae* and *Fusobacterium* and decreased *Akkermansia* (20) (18). Accumulating evidence indicates that the oral microbiota plays a significant role in this process, with specific oral pathogens acting as key contributors (**Table 1**). For instance, oral administration of *P. gingivalis* in mice results in elevated abundance of *Lachnospiraceae*, *Acetatifactor*, and *Acholeplasmataceae*, while *Rikenellaceae* and *Mycoplasmataceae* are more prevalent in the control group (21, 22). Beyond *P. gingivalis*, other oral pathogens also contribute to gut dysbiosis. Oral inoculation with *Streptococcus mitis* promotes the expansion of *Lactobacillus*, *Bacteroides*, *Staphylococcus*, *Clostridium*, and *Jeotgalicoccus* in treated mice (23). Identification of the key oral microbial species and the mechanisms by which they induce gut dysbiosis may enable the development of targeted therapeutic strategies. Such approaches could include pharmacological suppression or elimination of pathogenic species, as well as the introduction of antagonistic or beneficial bacterial strains. These interventions may help restore gut microbial homeostasis and support intestinal health.

**Table 1:** Changes of the gut flora influenced by the oral flora in epidemiological and animal experiments.

Experimental object	Sample	Molding	Changes in species abundance	Phylum	Family	Genus	Species	References
systemically healthy patients with periodontitis	feces		+		Erysipelotrichaceae	Porphyromonadaceae, Tannerella, Treponema, Lachnospiracea_incertae_sedis, Blautia		(18)
			–			Streptococcaceae, Veillonellaceae, Pasteurellaceae, Formosa, Mitsuokella		
C57BL/6J mice	cecal contents	Gavage periodontitis patients’ salivary flora	+	Porphyromonadaceae		Fusobacterium		(18)
DSS-mice	cecum content	Gavage periodontitis patients’ salivary flora and then maxillary second molars ligatured with 4–0 silk thread	+	Aerococcus				(142)
			–	Blautia, Helicobacter				
DSS-mice	cecum content	Gavage periodontitis patients’ salivary flora	+		Enterobacteriaceae and Bacteroidaceae, Christensenellaceae, Atopobiaceae, Erysipelatoclostridiaceae		Klebsiella spp.	(143)
			–	Firmicutes	Muribaculacae and Prevotellaceae, Butyricicoccaceae			
APP^swe^/PS1^ΔE9^ (PAP)transgenic mice	feces	Gavage periodontitis patients’ salivary flora	+	Bacteroidetes		Sutterella		(20)
			–	Firmicutes		Lactobacillus		
apoE^-/-^ mice	feces	silk sutures tied around the both sides of second maxillary molars	+	phyla Firmicutes			Gemell, Allobaculum, Barnesiella, Sporobacter	(19)
			–			Akkermansia, Barnesiella	Eubacterium	
BALB/cJ mice	cecal samples	*P. gingivalis* administered via oral gavage	+			Acholeplasmataceae		(22)
C57BL/6J mice	feces	injection of sonicated *P. gingivalis*	+		families Alcaligenaceae, Erysipelotrichaceae	Sutterella, Allobaculum	Faecalibaculum rodentium, Lactobacillus johnsonii, Lactobacillus reuteri	(115)
			–	Tenericutes, Proteobacteria	Dehalobacteriaceae, Ruminococcaceae	Bilophila, Dehalobacterium		
C57BL/6J mice	Cecal contents	*P. gingivalis* applied to the buccal surface of the maxillary gingiva	+	Bacteroidetes		Bacteroides, Barnesiella, Parabacteroidetes	Ruminococcaceae genera Clostridium IV, Flavonifractor, Pseudoflavonifractor	(144)
			–			Porphyromonadaceae, genus Coprobacter	Bifidobacterium, Desulfovibrio, A.muciniphila	
C57BL/6J mice	Cecal contents	*P. gingivalis* applied to the buccal surface of the maxillary gingiva + antibiotics Sulfatrim	+			Bacteroides, Barnesiella, Parabacteroidetes	Parvibacter caecicola	(144)
			–	Coprobacter		Desulfovibrio and Coprobacter	Acetifactor, Lachnospira, and Anaeroplasma	
ApoE^−/−^ mice	feces	*P. gingivalis* suspension given to gingival margin of the molars	+			Lachnospiraceae and Acetatifactor		(21)
			–			Rikenellaceae and Mycoplasmataceae		
C57BL/6 mice	feces	challenged orally with *S. mitis*, *S. salivarius*, *P*. *gingivalis* or *P*. *nigrescens*	+			*Bacteroides*, *Staphylococcus*, *Clostridium* and *Jeotgalicoccus*	*S*. *sciuri*, *B*. *massiliensis* and *B*. *thetaiotaomicron*	(23)
			–			Rikenellaceae and Mycoplasmataceae	*L. salivarius, T. sanguinis*, and *Clostridium celatum*	
DSS-mice	feces	Gavage with *P. gingivalis*	+	*Bacteroidetes*				(91)
			–	Firmicutes, Verrucomicrobia and Actinobacteria				
C57BL/6J mice	rectal stool	intraperitoneally administered *P. gingivalis*-LPS	–			Allobaculum		(145)

### 3.2 The gut microbiome: functions, dysbiosis, and its impact on oral microbiome

The gut microbiota, consisting of trillions of microbial cells, represents the largest microbial community in the human body (24). It comprises 6 main phyla: *Firmicutes*, *Bacteroidetes*, *Actinobacteria*, *Fusobacteria*, *Proteobacteria* and *Verrucomicrobia*. Among these, *Bacteroidetes* and *Firmicutes* together account for more than 90% of the total population (25). Through dynamic interactions with the host, the gut microbiota contributes to essential physiological functions in four key areas: 1) Metabolism. The phyla *Bacteroidetes* and *Firmicutes* cooperate to degrade indigestible carbohydrates and produce SCFAs (26). 2) Defense. The gut flora can resist bacterial colonization through nutrient competition (27) and the secretion of antibacterial products such as SCFAs, bile acids and bacteriocins (28). 3) The development of immune system. Germ-free mice exhibit disabilities in the development of mesenteric lymph nodes and Peyer’s patches, reduced numbers of CD4, CD8, and Foxp3 T cells, and lower production of secretory immunoglobulin A (sIgA) in B cells, leading to heightened susceptibility to pathogens like *Shigella flexneri* and *Salmonella typhimurium* (29). 4) Neuroregulation. The gut flora can secrete endocrine factors that influence neurotransmitter secretion, facilitating communication between the intestine and brain (30). Overall, the gut microbiota maintains a balanced and mutually beneficial relationship with the host in healthy states.

Dysbiosis of the gut microbiota is closely linked to intestinal disorders such as irritable IBS, IBD and CRC. In IBS patients, microbial diversity often decreases, with increases in *Proteobacteria*, *Lactobacillaceae*, *Enterobacteriaceae*, and certain *Clostridium* and *Ruminococcus* species, alongside reductions in *Faecalibacterium*, *Bifidobacterium*, and *Bacteroides* (31, 32). Similar shifts occur in IBD, including enrichment of *Veillonellaceae*, *Enterobacteriaceae*, and *Fusobacteriaceae*, and depletion of *Firmicutes* and *Bifidobacterium* (9, 33, 34). In CRC, both microbial diversity and richness are reduced, with specific pathogens such as *Fusobacterium nucleatum* (*F. nucleatum*), *Escherichia coli*, and enterotoxigenic *Bacteroides fragilis* implicated in carcinogenesis (35, 36).

Gut dysbiosis also influences the oral microbiota. In IBD patients, saliva shows increased phylum *Actinobacteria* and *Proteobacteria*, decreased *Bacteroidetes*, and higher relative abundance of genera *Streptococcus*, *Rothia*, and *Actinomyces* (37). Animal models of colitis, including *C. rodentium* infection or DSS treatment, similarly present increased *Betaproteobacteria*, *Lactobacillus* and *Spirochetes* in the saliva (38). Alterations in the oral microbiome are also evident in colorectal neoplasia. CRC patients exhibit increased oral microbial diversity, elevated *Fusobacteria*, *Bacteroidetes*, and *Firmicutes*, and reduced *Absconditabacteria* and *Proteobacteria* at phylum level (39, 40). At the genus level, *Bacteroides*, *Streptococcus*, and *Desulfovibrio* are enriched, while *Porphyromonas* and *Prevotella* are decrease (40). Consistent findings are observed in CRC mouse models, which show increased oral alpha diversity and reduced relative abundance of the genera *Bacteroides*, *Gemella*, and *Streptococcus* (41). Although these oral microbial shifts are clearly associated with intestinal disease, it remains unclear whether they are a cause or consequence. Understanding this relationship may enable the use of salivary microbiota as a predictive biomarker for gut disorders.

## 4 The connection between the oral and gut microbiomes

### 4.1 Bacteria

The composition of the oral and gut microbiota shares similarities at the taxonomic level. Both communities comprise the phyla *Firmicutes*, *Fusobacteria, Proteobacteria*, *Bacteroidetes* and *Actinobacteria* (8, 42). They also share genera such as *Streptococcus*, *Veillonella*, *Actinomyces*, and *Haemophilus*, with up to 125 overlapping species in composition (2). Despite this overlap, the relative abundance of specific taxa differs, and the oral microbiome exhibits significantly higher alpha diversity than fecal samples (43). This similarity may be partly explained by the frequent transfer of oral microorganisms to the gut under healthy conditions, although their overall contribution to gut microbial composition appears limited. One human study reported that microbes of oral origin accounted for 0.0–9.37% of the rectal microbiota, with notable species including *Streptococcus salivarius*, *F. nucleatum subspecies vincentii*, *Streptococcus parasanguinis*, and *F. nucleatum subspecies animalis* (44).

During intestinal diseases such as IBS, IBD, and CRC, the presence of oral-origin microbiota in the gut is altered (**Table 2**). Studies analyzing fecal samples from patients with ulcerative colitis (UC) and Crohn’s disease (CD) show that their gut microbiota more closely resembles oral microbial communities than those of healthy individuals; specifically, abundances of *Enterobacteriaceae*, *Fusobacteriaceae*, *Gemellaceae*, *Nisseriaceae*, *Proteobacteria* and *Veillonellaceae* increase, and *Bacteroidale*, *Eubacterium*, *Firmicutes* and *Lactobacillus* decrease (45). Analyses of colon tissue samples reveal more abundant pathogenic oral bacteria in patients than in healthy controls, which include *Aggregatibacter*, *Corynebacterium*, *Eubacterium*, *Fusobacterium*, *Gemella*, *Lactobacillus*, *Porphyromonas*, *Pseudomonas*, *Staphylococcus*, *Streptococcus* and *Veillonella* (46). Similar patterns are observed in CRC, where several oral taxa are detected in the gut. Notably, intratumoral enrichment of oral pathobionts such as *Leptotrichia buccalis* and *Filifactor alocis* is significantly associated with higher mortality risk (47). These findings suggest that the ectopic colonization of oral bacteria in the gut increases during IBD or CRC. This colonization may alter gut microbial composition and exacerbate disease progression.

**Table 2:** Changes of oral microorganisms in the gut under different diseases in human samples.

Disease	Sample	Changes in species abundance	Phylum	Family	Genus	Species	References
UC	feces	+			Rothia, Streptococcus, Prevotella, Porphyromonadaceae, Neisseria, Veillonella, Gemella		(146)
	Frozen full thickness colon specimens	+	Firmicutes		Porphyromonas, Prevotella, Gemella, Staphylococcus, Streptococcus, Abiotrophia, Granulicatella, Lactobacillus, Lactococcus, Peptostreptococcus, Selenomonas, Veillonella, Parvimonas, Eubacterium, Fusobacterium, Pseudomonas, Aggregatibacter, Corynebacterium	Staphylococcus sciuri, Staphylococcus aureus, Streptococcus anginosus.	(46)
		–	Bacteroidetes, Actinobacteria			Corynebacterium kroppenstedtii, Corynebacterium durum	
CD	feces	+			Prevotella, Porphyromonadaceae, Atopobium		(146)
	Frozen full thickness colon specimens	+	Firmicutes		Porphyromonas, Prevotella, Gemella, Staphylococcus, Streptococcus, Abiotrophia, Granulicatella, Lactobacillus, Lactococcus, Peptostreptococcus, Selenomonas, Veillonella, Parvimonas, Eubacterium, Fusobacterium, Pseudomonas, Aggregatibacter, Corynebacterium	Staphylococcus sciuri, Staphylococcus aureus, Streptococcus anginosus.	(46)
		–	Bacteroidetes, Actinobacteria			Corynebacterium kroppenstedtii, Corynebacterium durum.	
CRC	feces	+	Firmicutes, Fusobacteria			*F. nucleatum* spp., Parvimonas micra, Peptostreptococcus stomatis	(2)
	feces	+			Porphyromonas, Clostridiales, Fusobacterium, Parvimonas, Peptostreptococcus, Gemella, Prevotella, Solobacterium	P.stomatis, S.anginosus, S.koreensis, S. moorei	(147)
	feces	–			Bacteroides, Blautia, F.prausnitzii, Sutterella	Collinsella aerofaciens, Alistipes putredinis	(148)
	mucosa cancer specimens	+			Fusobacterium, Streptococcus, Peptostreptococcus		(40, 148)
		–	Proteobacteria, Fusobacteria				

### 4.2 Fungal microbiome

Fungi represent a minor component of the microbiome compared to bacteria, yet they play important functional roles. In healthy individuals, *Candida* is the most prevalent fungal genus in the oral mycobiota, constituting approximately 75% of the community; Other common genera include *Cladosporium*, *Aureobasidium*, *Saccharomyces*, *Aspergillus*, *Fusarium*, and *Cryptococcus* (48). During disease states, both the diversity and abundance of fungi shift in the oral and intestinal environments. For instance, a decreased oral fungal diversity and increase in the genus *Pichia* correlate with the severity of the oral lesions (49). Patients with periodontitis exhibit a broader range of fungal species in saliva, including *Candida parapsilosis* and *C. zeylanoides*, which are typically absent in healthy individuals (50). High abundance of *Candida* is associated with an altered oral ecology, such as lower pH, which contributes to dental caries (49). Fungi also interact with bacteria, amplifying microbial imbalance and disease progression. For example, in the oral cavity, *C. albicans* interacts with *Streptococcus oralis*, promoting biofilm formation, disrupting epithelial junctions, and increasing tissue invasion (51).

In the gut, the predominant fungi are *Ascomycota* and *Basidiomycota* at the phylum level, with *Saccharomycetes* at the class level, and *Saccharomycetales* at the order level (52). In IBD, the fungal microbiota becomes imbalanced, characterized by an elevated Basidiomycota/Ascomycota ratio, reduced *Saccharomyces cerevisiae*, and increased *Candida albicans*, *C. glabrata* and *C. tropicalis* (53). Notably, the abundance of *C. albicans* correlates with disease remission and relapse, while *C. tropicalis* interacts with anti-*Saccharomyces cerevisiae* antibodies, a known biomarker of Crohn’s disease (49). Some fungi are directly pathogenic and exacerbate disease through interactions with host cells. For example, orally derived *Candida famata* impairs mucosal repair and worsens IBD via the myeloid cell-specific type I interferon (IFN)-CCL5 axis (54). Similarly, CRC patients show notable alterations in the gut mycobiota, including depletion of *Aspergillus kawachii* and enrichment of *Aspergillus rambellii*, *Erysiphe pulchra*, and *Moniliophthora perniciosa* (55).

Under conditions resembling the healthy distal gut, characterized by low oxygen, simple sugars, and a diverse bacterial community, most fungi are unable to persistently colonize. The majority of intestinal fungi are transient and appear only when introduced via saliva or diet (56). For example, *Saccharomyces* became undetectable in stool when a *S. cerevisiae*-free diet was consumed (56). Orally derived fungi can contribute to gut dysbiosis. *Candida albicans* inhibits the growth of other fungi including *Aspergillus*, *Cladosporium*, and *Bipolaris* in the gut (57). Similarly, in immunosuppressed mice, *C. albicans* infection suppresses commensal saprophytic fungi like *Rhizopus*, *Mucor*, and *Penicillium* (58). Moreover, *Aspergillus rambellii* co-enriches with pro-tumorigenic bacteria like *F. nucleatum*, *Parvimonas micra*, and *Gemella morbillorum*, contributing to carcinogenesis (48). Notably, more frequent tooth cleaning has been shown to markedly reduce *C. albicans* levels in stool (56). Future research should further investigate how oral fungi influence gut microbial balance and explore therapeutic strategies targeting fungal-bacterial interactions.

### 4.3 Virome

Viruses have received relatively limited attention in microbiome research due to their small size and detection challenges. The human virome predominantly consists of bacteriophages alongside eukaryotic viruses. In subgingival plaques and saliva, the most abundant viral families are *Siphoviridae*, *Myoviridae*, *Podoviridae*, and *Herpesviridae* (59). In the fecal virome, *Podoviridae*, *Myoviridae*, *Siphoviridae*, *Autographiviridae*, and *Ackermannviridae* are commonly observed (59). These pervasive phages influence the abundance and phenotypic traits of commensal microbes, thereby affecting host health Studies involving murine norovirus indicate that enteric viruses can modulate immune responses without causing diarrhea (60). Although it remains to be determined whether a healthy or normal virome exists, it is clear that viral nucleic acid recognition contributes to intestinal homeostasis and enhances barrier function in health state.

Recent evidence indicates that shifts in the virome accompany oral and intestinal diseases. In periodontitis, patients exhibit significantly elevated levels of Epstein-Barr virus and cytomegalovirus, alongside a reduction in Siphoviridae within subgingival plaques (61). In IBD, bacteriophages associated with *Bacteroides fragilis* are more prevalent, while UC is characterized by enrichment of *Caudovirales* bacteriophages in the mucosal virome (54). Similarly, patients with premalignant colorectal adenoma show increased abundance of viral families such as Microviridae, Podoviridae_crAss-like, and Quimbyviridae (62). Although direct evidence linking the oral and gut viromes remains limited, several findings suggest a connection between oral viruses and intestinal disease states. In the oral cavity, an increased prevalence of human polyomavirus correlates with adenomatous polyposis, while higher rates of single and multiple beta-human papillomavirus infections, including oncogenic genotypes such as HPV5, are observed in CRC patients (63). This may reflect the translocation of viruses from the oral cavity to the gut. Under healthy conditions, HB1 and HB2 phage families, associated with the widespread Firmicutes phylum, have been detected in both oral and stool samples (64). During periodontitis, Pepyhexavirus appears to transfer from subgingival plaques to the gut. Moreover, trends in Alphabaculovirus, Marseillevirus, and Emaravirus abundance in subgingival plaques parallel those in the gut (59). *Bingvirus* in the gut positively correlates with *Cheoctovirus* from both subgingival plaques and saliva, while gut *Pamexvirus* is negatively associated with *Kenoshavirus* and *Pepyhexavirus* (59).

Phages play multiple roles in microbiome modulation. They can directly infect and reduce bacterial abundance. During disease states, interaction networks between phages and bacteria diminish in both oral and gut environments (65). *Torbevirus*, *Cafeteriavirus*, *Emaravirus*, *Karamvirus*, and *Mufasoctovirus* in saliva positively correlate with red-complex bacterial genera (59). Experimental evidence shows that in the presence of phage CLB_P3, deletion of *fliA* or *rfaL* genes in *E. coli* enhances biofilm formation, a change potentially linked to IBD (66). In CRC patients, viruses predicted to infect *Bacteroidaceae* exhibited a higher average relative abundance, whereas those targeting *Bifidobacteriaceae* show lower abundance (62). Although viruses are not primary pathogens in periodontitis or dental caries, their abundance changes under disease conditions. Future research should clarify the impact of oral viruses on the gut microbiome and further elucidate virus-bacteria interactions.

## 5 The ways and mechanisms explaining the connections between the oral and gut microbiomes

### 5.1 Mechanisms by which the oral microbiome affects the gut microbiome

**Figure 1:**
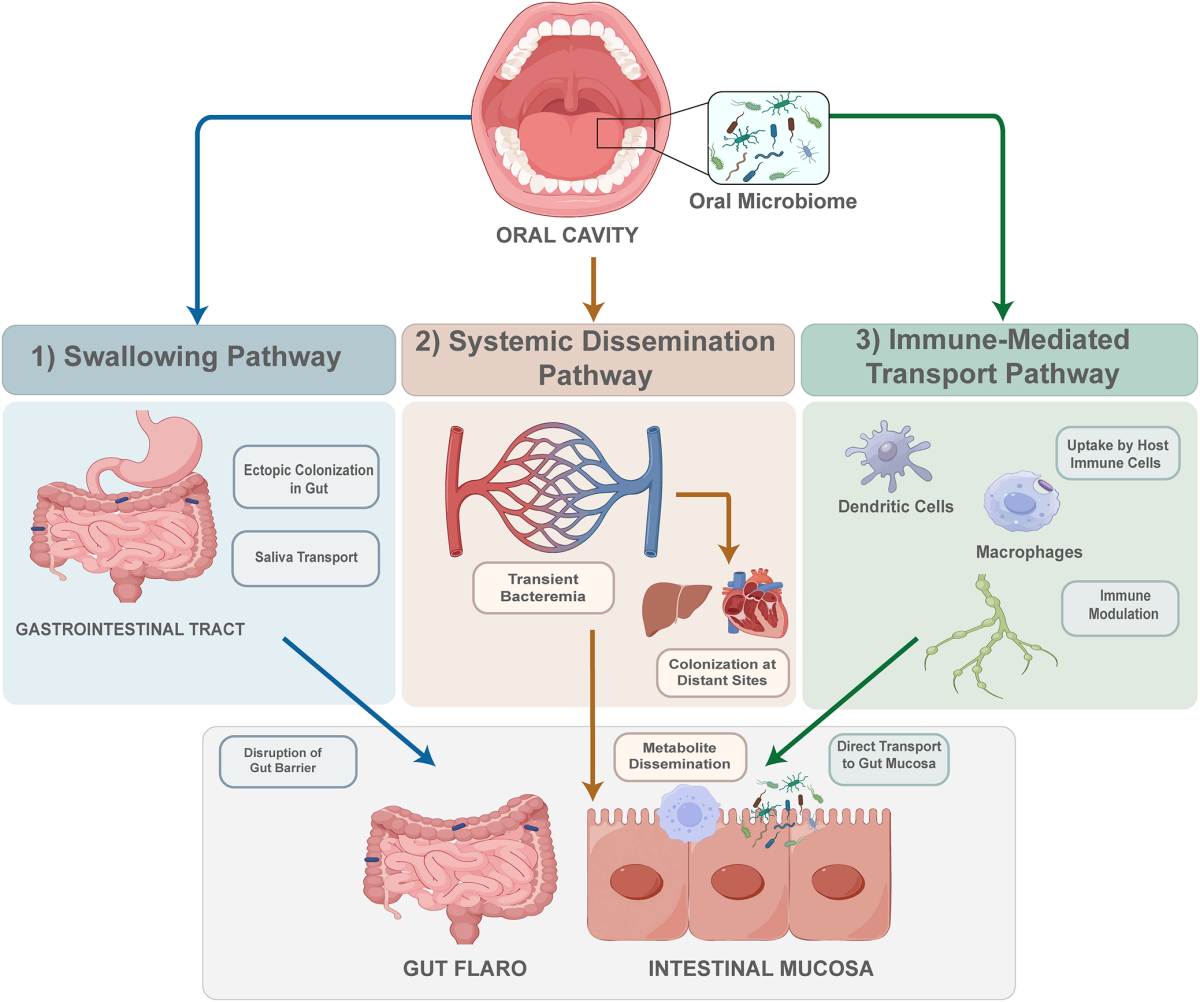
Three pathways of oral microbiome migration to the intestine. 1. Swallowing pathway. Oral microbiome from the oral cavity can transport via saliva to the gastrointestinal tract, leading to ectopic colonization in the gut and disruption of the gut barrier. 2. Systemic dissemination pathway. Inflammation in periodontal tissue leads to transient bacteremia and dissemination of metabolites, facilitating colonization at distant sites such as the liver, heart, and intestines. 3. Immune-mediated transport pathway. Oral microbes are taken up by host immune cells like dendritic cells and macrophages, modulating immune responses and promoting direct transport to the gut mucosa, affecting intestinal health.

The microbiological pathologic link between oral and systemic diseases is becoming a widely discussed topic. Current research generally suggests that oral flora can influence gut flora through the following pathways (67):1)Swallowing. The oral microbiome and its metabolites travel through the gastrointestinal tract to the gut tract and impact the intestinal microbiome. 2) Systemic dissemination. Oral microbes and their byproducts may enter the systemic circulation, leading to transient bacteremia and potential colonization at distant sites. 3) Immune-mediated transport. Through direct infection, oral microorganisms can be taken up by host immune cells, such as dendritic cells and macrophages, and transported to the intestinal mucosa without systemic circulation (**Figure 1**).

**Figure 2:**
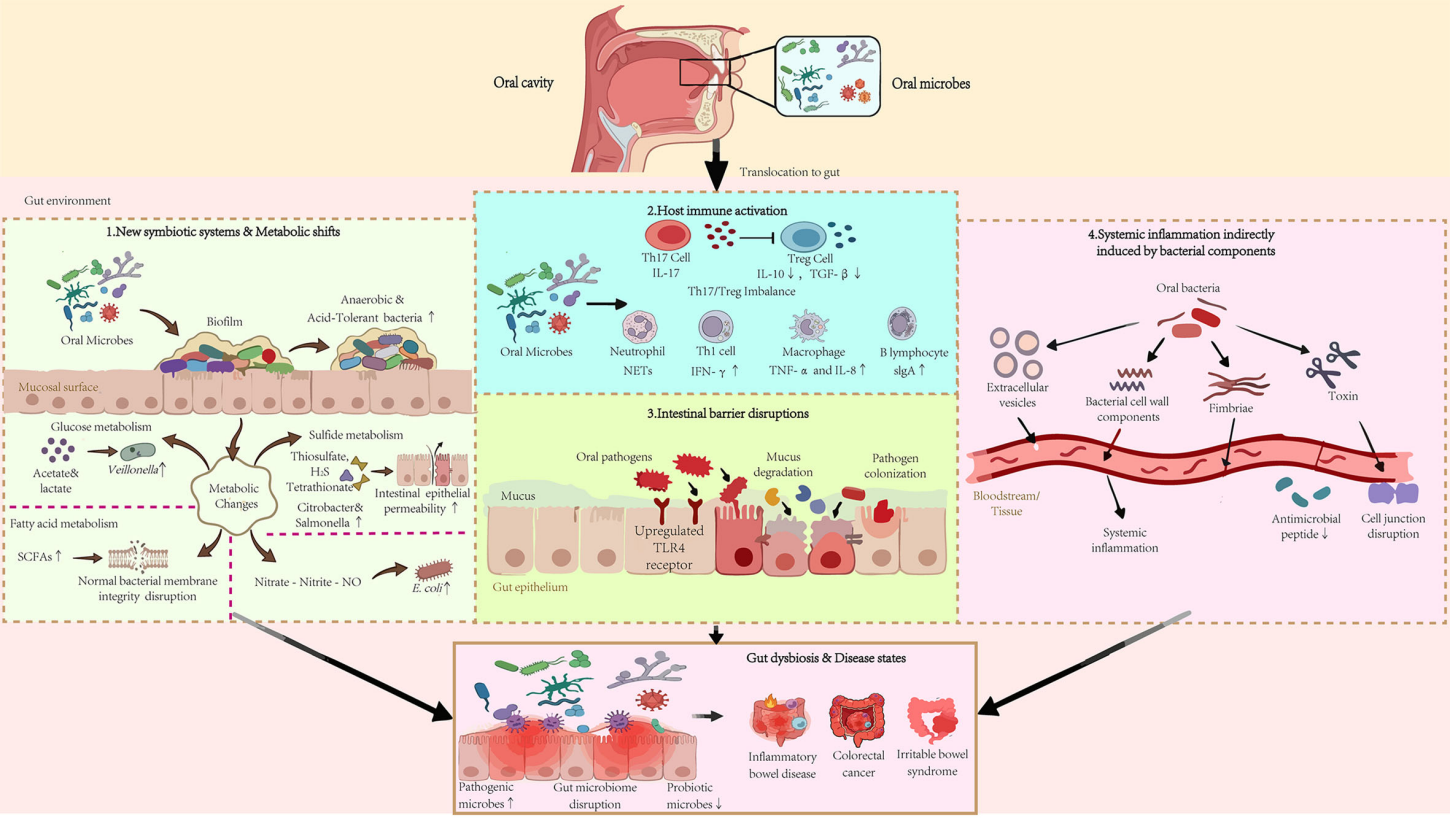
Four mechanisms by which oral microbiome induce gut dysbiosis. This figure illustrates the complex interactions between oral microbes and the gut environment leading to gut dysbiosis. The translocation of oral microbes to the gut triggers various effects. 1. New symbiotic systems formation and metabolic shifts. Oral microbes adhere and aggregate on the mucosal surface, forming biofilms that promote anaerobic and acid-tolerant bacteria. This leads to metabolic changes of glucose, fatty acid, sulfide and nitrate, disrupting normal bacterial membrane integrity and promoting proliferation of *E. coli* and other pathogens. 2. Host immune activation. Oral bacteria affect immune cells, causing a Th17/Treg imbalance and highlighting inflammation pathways involving neutrophils, Th1 cells, macrophages, and B lymphocytes. 3. Intestinal barrier disruption. Oral pathogens degrade mucus and colonize by upregulating TLR4 receptors on gut epithelium, leading to epithelial barrier damage. 4. Systemic inflammation indirectly induced by bacterial components. Oral bacteria release components including EVs, lipopolysaccharides (LPS), fimbriae, and gingipains into the bloodstream and tissue, causing systemic inflammation and tissue damage through immune evasion and disruption of cell junctions.

However, the specific mechanisms by which oral microbes shape the composition of the gut microbiota remain unclear. Based on current evidence, we summarize these mechanisms into the following broad categories (**Figure 2**):

#### 5.1.1 The oral microbiota can establish new symbiotic systems and metabolic correlations with the gut microbiota

Oral microorganisms are capable of forming stable symbiotic systems within biofilms. UAfter translocation to the gut, these organisms may establish novel symbiotic networks with the resident gut microbiota. Such newly formed symbiotic systems can potentially protect orally derived pathogens. For instance, *P. aeruginosa*, *Pseudomonas protegens*, and *Klebsiella pneumoniae* can form a multispecies biofilm that synergistically degrades a toxin while none of the species can metabolize independently (68). The emergence of these cooperative microbial networks can substantially modify the local gut microenvironment, thereby selecting for taxa adapted to the altered conditions. Similarly, colonization of *P. stomatis* contributes to the acidic environment by producing acetic, isobutyric, isovaleric, and isocaproic acids (69). This acidic milieu may favor the enrichment of acid-tolerant bacteria within the gut ecosystem. Certain acid-tolerant bacteria like *E. coli* and *Propionibacterium acidipropionici*, possess multiple adaptive strategies to withstand low pH levels and maintain their stationary phase. These mechanisms include membrane remodeling, activation of proton extrusion systems, biofilm formation, alkali production, and enhanced protection or repair of macromolecules, which collectively allowing such bacteria to withstand environmental stress and become dominant members of the reshaped gut microbiota (70, 71).

Viruses and fungi also participate in symbiotic networks through interactions with bacteria. Antibiotic resistance genes can be transferred between bacteriophages and bacteria. In addition, virulence factors such as the adhesin YadA from *Yersinia enterocolitica* may be disseminated through phage-bacteria interactions, thereby enhancing bacterial adhesion and persistence (72). These horizontal gene transfer events increase bacterial pathogenicity and resilience, contributing to microbial imbalance. Increased abundance of *Candida albicans* in the gut is related to reduced levels of bacterial genera such as *Ralstonia*, *Alistipes*, *Clostridia*, *Ruminococcus*, and *Lachnospiraceae (*57). Particularly, under immunosuppressed conditions, extensive colonization by orally derived *C. albicans* leads to the markedly depletion of beneficial bacteria such as *Bifidobacterium*, *Dubosiella*, and *Turicibacter (*58). In contrast, pathogenic bacteria including *Clostridium*, *Escherichia coli*, and *Enterococcus* become enriched (58). Furthermore, physical interaction between *C. albicans* and bacteria like *E. faecalis* promotes their accumulation on host mucosal cells (73). Additionally, a synergistic relationship between *C. albicans* and *Enterococcus faecalis* has been demonstrated. *E. faecalis* degrades the epithelial junction protein E-cadherin, facilitating *C. albicans* invasion in an *in vivo* oropharyngeal candidiasis infection model (74).

On the other hand, colonization of oral microbiota can also bring new metabolic partnerships into the gut ecosystem. This process increases the number of microorganisms involved in previously absent metabolic interactions. For example, in carbohydrate metabolism, oral bacteria such as *Lactobacillus* spp. and *Streptococcus* may compete with the host for sugar uptake in the gut. These bacteria degrade complex sugars into acetate and lactate, which can in turn serve as metabolic substrates for *Veillonella*, a potential pathogen linked to IBD (75). In fatty acid metabolism, the enrichment of oral anaerobes such as *Veillonella* and *P. gingivalis* promotes the fermentation of carbohydrates and proteins into SCFAs like acetate, propionate, and butyrate (76). These SCFAs can disrupt bacterial membrane integrity and subsequently inhibit biofilm formation by beneficial commensals. Oral bacteria also contribute to the metabolism of glycerophospholipids, LPS, and phosphonoacetate, supplying alternative carbon and phosphate sources to other gut microbes (76). For the nitrate-nitrite-NO pathway, anaerobic oral bacteria generate bioactive NO after entering the gut (46). A nitrate-rich environment benefits the multiplication of *Enterobacteria* like *E. coli*, which possess genes encoding nitrate reductases (77). Alterations are also evident in sulfur metabolism. Patients with CD exhibit increased abundance of oral-derived bacteria like *Rothia dentocariosa*, *F. nucleatum*, and sulfidogenic microbes like *Prevotella copri* and *Bilophila* spp (78). Metagenomic analyses reveal dysregulation of cysteine and methionine metabolism, ATP transport, and redox pathways, indicating disturbed sulfur metabolism (78). These changes result in elevated hydrogen sulfide levels in the gut. Hydrogen sulfide disrupts disulfide bonds in mucin polymers, reduces mucus viscosity, and increases intestinal permeability. As a consequence, colonization by beneficial bacteria is impaired (79). Together, these emerging symbiotic relationships suggest that oral microbes and their metabolites may contribute to intestinal disease directly or indirectly by destabilizing gut microbial homeostasis. Unraveling these interactions could enable microbiota-based interventions, including targeted probiotics or metabolite supplementation, to restore ecological equilibrium and promote intestinal health.

#### 5.1.2 The oral microbiome affects intestinal immunity, thus promoting inflammation and interfering with gut flora homeostasis

After translocation to the intestine, oral pathogenic microbes interact with antigen-presenting cells, particularly dendritic cells. This interaction triggers the release of large amounts of inflammatory cytokines. The resulting immune activation promotes the differentiation of T cells and activation of neutrophil, initiates local inflammation, and ultimately exacerbates gut dysbiosis as well as intestinal tissue damage. For example, periodontal pathogens can recruit inflammatory cells like neutrophils (80). *F. nucleatum* activates toll-like receptors 4 (TLR4), inducing the generation of reactive oxygen species (ROS) within neutrophils. ROS act as downstream signaling molecules that drive the release of neutrophil extracellular traps (NETs). Concurrently, *F. nucleatum* upregulates intracellular receptors NOD1 and NOD2. The NOD1/2 signaling pathway, which operates independently of ROS, further contributes to the regulation of NET formation (81). Infection with *Streptococcus* strains TW289 and ATCC10556 activates a Th1-biased immune response, increasing IFN-γ secretion by T cells and thereby promoting inflammatory aggravation (82). Some oral strains of *Campylobacter concisus* carry the zonula occludens toxin gene acquired from prophages. Through zonula occludens toxin, *Campylobacter* activates inflammasome signaling in macrophages, elevating the expression of tumor necrosis factor α (TNF-α) and interleukin (IL)-8 (83). In addition, *Klebsiella pneumoniae* isolated from the salivary microbiome of patients with CD, particularly strain Kp-2H7, activates epithelial cells and dendritic cells via the TLR4 pathway. This activation stimulatesIL-18 secretion and promotes the recruitment and activation of Th1 cells, thereby amplifying intestinal inflammation (84).

The activation of immune cells and the release of inflammatory factors disrupt the balance between host cells and the gut microbiota. In response to cytokines such as IL-17 and IL-22, antimicrobial peptides are produced, which in turn alter the composition of the gastrointestinal microbiota. Similarly, IgA-producing B lymphocytes generated in Peyer’s patches and mesenteric lymph nodes mature into plasma cells. These cells accumulate in the lamina propria and regulate gut microbial populations through the secretion of immunoglobulin A (IgA), thereby limiting the expansion of harmful bacteria (85). Intestinal inflammation creates a microenvironment that favors the expansion of *Enterobacteriaceae (*86). Elevated levels of inflammatory cytokines, including IFN-γ and TNF-α, also drive alterations in host metabolic pathways, notably enhancing tryptophan catabolism via the kynurenine pathway. This metabolic shift further influences the gut microbial community, promoting dysbiosis that is frequently associated with IBD and IBS (87).

Numerous studies have found that the balance between Th17 cells and Treg cells is closely associated with microbes and inflammation. After oral bacteria invade the gut, microbiome DNA, Candidalysin from *C. albicans*, *C. difficile* toxins, and proinflammatory cytokines such as IL-1β and TNF-α, along with chemokines like MIP-1α and IL-8 released by innate immune cells, promote the differentiation of CD4^+^ T cells into Th17 cells (88). Th17 cells secrete IL-17, which exacerbates inflammation and favors the growth of bacteria adapted to inflammatory states. Concurrently, Th17 proliferation suppresses the differentiation of Tregs mediated via TLR9, thereby impairing the anti-inflammatory functions of IL-10 and transforming growth factor-β (89). Reduced IL-10 levels are associated with increased abundances of *Bacteroidetes*, *Escherichia coli*, *Proteobacteria*, and *Verrucomicrobia (*89). Impaired TGF-β signaling further promotes the enrichment of *Enterobacteriaceae*, particularly *E. coli (*89). However, conflicting evidence has also been reported. For example, mice treated with *P. histicola* presented decreased IL-17 and TNF-α levels, low antigen-specific Th17 responses and enriched Treg cells in the gut (90). This suppression of Th17 activity may be related to the ability of oral pathogens such as *P. gingivalis* to inhibit the linoleic acid metabolic pathway in the gut microbiota. Linoleic acid is an anti-inflammatory metabolite that suppresses Th17 differentiation via activation of the aryl hydrocarbon receptor and promotes Treg differentiation through Stat1 phosphorylation at Ser727 (91). When Th17 responses are suppressed, host clearance of gut microbes may weaken, potentially allowing pathogenic overgrowth. Indeed, IL-17 deficiency has been linked to increased Proteobacteria and Bacteroidetes and decreased *Akkermansia muciniphila* in the gut microbiome (92). Together, these findings indicate that both excessive and insufficient inflammation can disrupt intestinal homeostasis. These opposing immune states may occur at different anatomical sites or disease stages. Nevertheless, it is evident that oral microorganisms can disturb the dynamic balance between host immune regulation and the gut microbiota, ultimately leading to dysregulated inflammation.

Beyond bacteria, fungi and viruses also interfere with host immune responses. *Candida albicans* can infect host cells and activate multiple signaling pathways, including PI3K/Akt/NF-κB, p38/JNK, and ERK1/2-MAPK. This activation promotes the release of antimicrobial peptides and pro-inflammatory cytokines, contributing to a sustained inflammatory state (93). During invasive *C. albicans* infection, the secreted protein Sel1 further triggers a TLR2/4-dependent inflammatory response, activating both NF-κB and MAPK pathways and inducing the expression of proinflammatory cytokines and chemokines (93). Similarly, viral infections such as cytomegalovirus modulate immune defenses in ways that may benefit bacterial pathogens. For instance, cytomegalovirus enhances neutrophil chemotaxis and inhibits neutrophil apoptosis, potentially facilitating the invasion and persistence of oral bacteria such as *Aggregatibacter actinomycetemcomitans* and *P. gingivalis* in epithelial tissues (94). In cytomegalovirus-infected epithelium, the production of innate immune mediators is markedly increased. Elevated levels of IL-1β and TNF-α are observed, along with enhanced expression of chemokines including IL-8, monocyte chemoattractant protein-1, macrophage inflammatory protein-1α, and macrophage inflammatory protein-1β (94).

Some oral bacteria have the capacity to inhibit immune defenses and evade host immune surveillance, allowing them to thrive as pathogens. Pathogenic bacteria can directly interact with immune cells, inhibiting their activation and killing effects. *Fusobacterium* can inhibit T-cell responses and suppress cell-mediated immunity and phagocyte functions (95). Colonization of the oral flora leads to increased SCFAs production in the gut, which can inhibit histone deacetylase activity, resulting in highly acetylated histones that regulate gene expression related to inflammation processes or induce growth inhibition and apoptosis in associated cells, thereby generating immune tolerance (96). Other bacteria avoid being cleared by immune cells by breaking down signaling molecules or immune proteins. Several species within the genus *Gemella* can cleave IgA1, resulting in significant proliferation of anaerobic bacteria, particularly mucosa-adherent segmented filamentous bacteria belonging to the phylum *Firmicutes*. Beyond bacteria, oral bacteriophages may also contribute to immune evasion. Virulence genes carried by these phages, such as *pspA* and *pspC*, are implicated in complement interference and immunoglobulin degradation (72).The balance between the normal intestinal flora and intestinal immune cells may be disrupted due to the ability of these oral bacteria to suppress immune defenses, resulting in the proliferation of specific pathogens and a reduction in microbiome diversity (97).

#### 5.1.3 Oral microbiome damages the epithelial barrier interfering with gut flora homeostasis

The oral microbiota may change the expression of surface receptors on intestinal epithelial cells, thus promoting bacterial colonization. Through the interaction between increased TLR4 in epithelial and lamina propria cells of IBD patients and the pili-like external membrane protein Amuc-1100, the establishment of *A. muciniphila* might be improved (98). In colonic epithelial cells and dendritic cells colonized with *K. pneumoniae*, I*F. NUCLEATUM*-inducible genes, such as those programming guanylate-binding proteins, are significantly upregulated (84). These genes act as microbial receptors and may promote the adsorption and colonization of microorganisms (99). Oral microbes can also alter the inflammatory response of the intestinal epithelium. The periodontal pathogen-associated metabolite isoleucine enhances the NF-κB signaling pathway in intestinal organoids and IEC-6 cells, intensifying inflammatory responses (80). *F. nucleatum* and *P. gingivalis* jointly stimulate intestinal epithelial cells to release inflammatory factors such as IL-8, thereby creating a pro-inflammatory microenvironment (100).

The disruption of epithelial cells may alter the substrate source of microorganisms and promote the proliferation of species adapted to the new environment. The intestinal epithelium can be damaged by *F. nucleatum* via a molecular network involving CARD3 and IL-17F (86) and by *P. gingivalis* through CD4 T cells that produce IL-9 (22). Experimental periodontitis and periodontitis-associated metabolite isoleucine increased the intestinal permeability, downregulated the expression of tight junctions including ZO-1 and occludin (80). The oral administration of *A. actinomycetemcomitans* was followed by increased expression of pro-inflammatory cytokines like TNF-*α*, IL-6 and IL-1β, concurrent with reduced expression of barrier proteins like ZO-1 and MUC-2 and alterations in the composition of the intestinal flora in mice with colitis (101). Beyond bacteria, fungi also contribute to epithelial injury. *Candida albicans* invades host mucosal layers via endocytosis or active penetration, subsequently inducing epithelial apoptosis and necrosis (102). Indirect damage may also occur through candidalysin, a cytolytic peptide toxin secreted during hyphal growth (58). Furthermore, glucose depletion caused by *C. albicans* sensitizes enterocytes to bacterial cytolysins, amplifying epithelial damage (73).

The mucus secretion of goblet cells impaired by ectopic bacteria can favor the proliferation of species that are able to use mucin glycans as metabolic substrates. Enterotoxigenic *Bacteroides fragilis* strains can promote mucin degradation, favoring colonization by *E. coli (*103). Epithelial dysfunction reduces secretion of the antimicrobial peptide Reg3γ, whose loss exacerbates colitis, impairs clearance of *Bacteroides acidifaciens*, and alters the abundance of *Akkermansia muciniphila (*104). Identifying the mechanism of microbial influence on cells and inhibiting the influence process can enable cells to continue maintaining normal function and resist microbial invasion. It is undeniable that local host cell damage caused by oral pathogenic bacteria may be one of the causes of intestinal flora imbalance.

#### 5.1.4 Oral microbes release virulence components to induce systemic inflammation and disrupt the balance of the gut microbiota

In some cases, the DNA of oral pathogens cannot be directly detected in the intestine (22). However, bacterial components such as LPS, peptidoglycan, or lipoteichoic acid may enter the bloodstream and induce endotoxemia. Animal models of periodontitis have shown elevated serum levels of neutrophils, acute-phase proteins such as C-reactive protein and serum amyloid A, and inflammatory cytokines including IL-1β and IL-6 (105). These findings suggest that, beyond direct interactions with gut bacteria or host cells, oral pathogens can also influence systemic and intestinal homeostasis through the release of specific structural molecules or secreted factors.

1. Extracellular vesicles. The ratio of cells to extracellular vesicles is approximately 1:2000. Compared to whole cells, extracellular vesicles penetrate epithelial barriers more efficiently and participate in immune regulation by carrying peptidoglycans, lipids, proteins, and nucleic acids (106). *P. gingivalis*-derived extracellular vesicles can interact with RgpA and EGFR to activate the PI3K-AKT pathway in dendritic cells, increasing the production of IL-6, TNF-α, and IL-1β (107). Additionally, they can activate neutrophils, triggering degranulation without causing cell death and thereby promoting inflammation (108). Neutrophils contribute to the elimination of microorganisms through phagocytosis, degranulation, the generation of ROS, and the release of NETs, leading to microbial imbalance (109).
2. Bacterial cell wall components. Oral microorganisms like *P. intermedius* have the ability to generate harmful substances such as LPS, peptidoglycan, or lipoteichoic acid. These substances prompt the production of proinflammatory cytokines (110). *Segatella Copri* R-LPS may act as a competitive ligand for the TLR4/MD-2 complex, inhibiting TLR4-dependent NF-κB activation induced by pro-inflammatory *E. coli*-LPS, and thereby dampening host inflammatory responses (111). Other bacterial cell wall components, such as peptidoglycan subunits, have been shown to promote filamentation and invasive infection by *Candida albicans* in the gastrointestinal tract (112). This inflammatory state can alter the composition and reduce the diversity of the gut microbiome (109).
3. Fimbriae. Co-culture of *P. gingivalis* with *Streptococcus gordonii* and *F. nucleatum* upregulates the expression of Mfa1 fimbriae, which enhances dendritic cell infection while inhibiting their maturation (113). Long filamentous structures like the surficial pilus of Streptococcus and amyloid-like fusobacterium adhesion A produced by *Fusobacterium* under stress and disease conditions, exhibit adhesive properties and can promote the aggregation of bacteria and biofilm thickening, creating an anaerobic environment in deep biofilms (114). This could be a possible explanation for the increase of anaerobic bacteria in the intestines of *P. gingivalis*-injected mice (115).Furthermore, *F. nucleatum* infiltrates the intestinal epithelial tissue using specific outer membrane proteins such as fusobacterium protein A, fusobacterium adhesion A, and fusobacterium self-transporter 2. This invasion triggers the activation of signaling pathways involving NF-κB and TLR4, promoting inflammation (110). The activation of the TLR4 pathway is closely related to the abundance of intestinal microbiota. The relative abundance of Lactobacillus and the number of Paneth cells remarkably decreased in TLR4-deficient mice (116).
4. Toxin. LtxA, a toxin produced by *Aggregatibacter actinomycetemcomitans*, acts as a virulence factor for A. actinomycetemcomitans to subvert the host immune response by binding to the β_2_ integrin lymphocyte function-associated antigen-1 (LFA-1; CD11a/CD18) on white blood cells, causing cell death (117). *P. gingivalis* culture supernatants contain gingipains, which can fully or partially degrade human α- and β-defensins, key antimicrobial peptides in the intestinal mucosa (118). *In vitro* studies show that gingipains cleave PECAM-1, VE-cadherin, and E-selectin on human endothelial cells, resulting in loss of cell–cell contacts, impaired adhesion, and eventual cell death (119). By interfering with host cells through these components, oral pathogens can induce immune dysregulation, potentially transforming commensal gut microbes into pathogenic entities. For instance, *Mucispirillum schaedleri* suppresses *Salmonella* virulence in immunocompetent hosts but can act as a pathobiont and induce colitis in immunocompromised hosts (120).

### 5.2 Mechanisms by which the gut microbiome affects the oral microbiome

#### 5.2.1 Migration of cytokine and the immune cells to the mouth

Under normal circumstances, it is difficult for the intestinal flora to be directly transferred to the mouth, so its impact on the oral flora is more indirect through the host immune system. Gingival inflammation in IBD patients does not always correspond with plaque accumulation (121). This may be explained by the shared mucosal immune system connecting the oral and intestinal mucosae. Intestinal inflammation can alter the host’s immune response toward oral bacteria, promoting oral dysbiosis and exacerbating local inflammation (122). Supporting this, the gut genus *Alistipes* has been identified as a risk factor for periodontitis, while fractalkine may serve as an inflammatory mediator linking gut Actinobacteria to periodontal disease (123). In IBD, dendritic cells beneath the intestinal epithelium produce elevated levels of cytokines such as IL-1β, IL-6, IL-18, IL-23, and TNF, which are also elevated in saliva (124). *Actinomyces*, *Lachnospiracea*, *Prevotella* and *Veillonella* are positively associated with elevated cytokine levels in saliva, while *Gemella*, *Haemophilus*, *Neisseria*, *Rothia* and *Streptococcus* tend to be negatively correlated (125). Except for cytokines, gut-translocated *P. gingivalis* can reach Peyer’s patches through M cells and trigger gut-originated Th17 cells to migrate to the oral cavity (126). In periodontal tissues, upregulation of the chemokine CCL20, which binds to CCR6 on Th17 cells, likely facilitates Th17 accumulation locally (126). Th17 cells expansion, dependent on IL-6 and IL-23 signaling, promotes tissue destruction and may enhance colonization of oral pathogens within epithelial niches (127). The role of this common mucosal immune response remains to be explored. The migration of inflammatory factors and inflammatory cells to the mouth may clear oral pathogens and reduce their influence on the gut, or it may disrupt the oral microbial balance and bring new problems.

#### 5.2.2 Malnutrition

Owing to microbiota dysbiosis and inflammatory processes in the gut, the loss of normal absorption pathways and higher nutrient requirements can cause malnutrition, resulting in deficiencies in enzymes and proteins that maintain oral homeostasis and impairing the host’s innate and adaptive defenses (124). Zinc deficiency disrupts Gustin CAVI, a zinc-dependent enzyme that adjusts saliva pH, increasing the acidity of the oral environment (128), which can favor the growth of *S. mutans*, *Lactobacillus* and *Candida* species (129). In protein energy malnutrition, there is also dysfunction of the cytokine system and a reduction in acute-phase protein reactions. Lysozyme, which is responsible for hydrolyzing the gram-positive bacterial cell wall and can also kill gram-negative bacteria when working with salivary lactoferrin, is significantly decreased in the saliva of IBD patients (125). As a result, pathogenic bacteria in the mouth of IBD patients are more likely to multiply than are those in healthy individuals.

#### 5.2.3 Diet

By modulating taste receptor expression, the gut microbiome and its metabolites may modify the diet and indirectly influence the oral microbiome. Although there is no systematic explanation of the specific mechanism by which dysregulation of the gut flora leads to altered taste, the association between bacteria and taste receptors has been studied. All taste sensitivities, with the exception of sour taste, are significantly reduced in IBD patients (128). It has been reported that gut microbiota-derived tryptophan metabolites can act as agonists of bitter taste receptors (130), and responses to natural and artificial sweeteners are suppressed in DSS-treated mice (131), leading to an increase in the host’s intake of sweet foods. High sugar intake may lead to dental caries and a substantial reduction in oral microbiome diversity and the accumulation of several bacteria, including *Actinomyces*, *Lactobacillus*, *Rothia*, *Scardovia*, *Streptococcus* and *Veillonella (*132).

## 6 Treatment and application

### 6.1 Treatment targeting microbiome balance

Based on the above-mentioned connection between oral microbiome and gut microbiome, treating oral diseases may help restore gut microbial balance and alleviate intestinal disorders. In mice, nonsurgical periodontal treatment can restore gut barrier function and microbiota composition, promoting the recovery of beneficial genera like *Turicibacter* and *Bifidobacterium* while reducing *Gemella*, *Allobaculum*, and *Barnesiella (*19). Similar observations have been reported in human patients. After undergoing Steps I-II of periodontal therapy, periodontitis patients exhibited a decrease in stool pathogens—including *Clostridium perfringens*, *Campylobacter concisus*, and *Klebsiella pneumoniae*—as well as oral-origin taxa such as *Eikenella corrodens*, *F. nucleatum*, and *Porphyromonas endodontalis*. Concurrently, there was an increase in short-chain fatty acid-producing species like *Akkermansia muciniphila* and *Bifidobacterium breve (*133). When administering dental antibiotics to patients with IBD, caution is warranted. First and second line dental antibiotics such as amoxicillin, ampicillin, and clindamycin, carry an elevated risk of *Clostridioides difficile* infection in this population (134).

Other approaches that simultaneously modulate both oral and gut microbiomes also show promise. For instance, regular use of mouthwash was associated with oral microbial shifts, including reductions in *Gemella haemolysans*, *Streptococcus oralis* and *Granulicatella* sp., along with increases in *Actinomyces viscosus*. At the same time, taxonomic shifts in the gut included enrichment of the *Bacteroides*, *Phocaeicola* and *Alistipes* species, and decreases in *Lachnospiraceae*, *Intestinibacter* sp. and *Blautia luti (*135). Consumption of kefir, a fermented probiotic beverage, has been linked to increased levels of *Lactobacillus* in individuals with IBD, correlating with improved gastrointestinal symptoms. Studies also reported reduced colony-forming units of salivary *Streptococcus mutans*, a key contributor to dental caries, following kefir intake (136). Moving forward, integrated management for oral and gut microbial balance should be considered in the prevention and treatment of intestinal diseases. Targeted oral hygiene interventions and dietary probiotics may offer dual benefits for maintaining microbial homeostasis and supporting gastrointestinal health.

### 6.2 Biomarkers for gut diseases

Current gold standard strategies for diagnosis and monitoring such as endoscopy and biopsy are invasive, costly, burdensome. Non-invasive screening approaches, including fecal immunochemical tests and guaiac-based fecal occult blood tests that detect the presence of hidden blood in stool samples, are limited by suboptimal sensitivity and specificity (137). Given the established associations between the oral microbiome and intestinal disorders, specific oral microbial signatures could serve as promising biomarkers for detecting gut diseases and assessing their severity. Saliva collection offers a non-invasive, convenient, and repeatable alternative. For example, the Optimized Salivary MetaProteomic sample analysis workflow significantly enhanced the identification of bacterial peptides and proteins by 3.2 folds and 1.7 folds compared to conventional approaches. In saliva of IBD patients, proteins involved in the fatty acid elongation pathway of *Peptostreptococcus* were significantly reduced, while TCA cycle-associated proteins from *Neisseria* were notably elevated (138). Moreover, receiver operating characteristic curve analysis illustrated that *F. nucleatum* DNA was superior to carcinoembryonic antigen and carbohydrate antigen 19–9 in CRC diagnosis. Levels of salivary *F. nucleatum* DNA also correlated with the overall survival and disease-free survival of CRC patients, which was an independent factor for prognostic prediction (139).

Beyond microbiome markers, other salivary molecules such as proteins, microRNA show predictive potential. In CD patients, oxidative stress markers like malondialdehyde are significantly elevated, while in UC, NO levels rise to approximately four times the normal range (140). Additionally, a panel of five salivary miRNAs, miR-186-5p, miR-29a-3p, miR-29c-3p, miR-766-5p, and miR-491-5p, could differentiate CRC patients across stages I–IV from healthy individuals with a sensitivity of 72.0% and specificity of 66.67% (141).

However, there is currently insufficient evidence to demonstrate the reliability and specificity of salivary biomarkers in predicting intestinal diseases. On one hand, the composition of saliva is influenced by multiple confounding factors, including oral hygiene, dietary habits, oral medications and circadian rhythms, which introduce significant uncertainties into its detection results. Moreover, existing model studies generally lack comprehensive evaluations of volunteers’ oral health conditions due to the absence of standardized protocols for saliva collection and established reference ranges for microbial abundance. As a result, the reliability of the current evidence is weakened. On the other hand, most studies only report changes in salivary biomarkers during intestinal diseases without conducting reverse deduction or validation. Consequently, it remains unclear whether alterations in salivary biomarkers specifically indicate a particular disease or merely reflect a systemic inflammatory state. Future research is needed to refine salivary sampling methods, validate salivary biomarker panels, and define clinically applicable thresholds for oral microbiome-based diagnostics in gut diseases. Furthermore, multicenter, large-scale clinical research should be conducted to prospectively explore the potential of salivary biomarkers in intestinal and systemic diseases.

## 7 Conclusion and perspectives

The presence and importance of oral and intestinal microbiota are gradually being recognized, and there are also interactions between them, especially when diseases such as periodontitis and intestinal inflammation occur. However, most studies exploring the pathological links between the oral and gut microbiomes are observational and are still in the association stage. Experiments that investigate the cellular and molecular mechanisms behind such changes are needed. The establishment of new symbiotic relationships, the activation and suppression of inflammatory cells, and the systemic inflammation state may be used to explain their associations. Elucidating the mechanism of action could help develop new methods for the prevention, treatment and monitoring of intestinal diseases that target the gut microbiome. It can also provide insights for a better understanding of the associations between oral and systemic diseases.
